# Supplementary material for: Spurious Asthma Presentation during COVID-19
Source: Children (Basel). 2021 Dec 23;9(1):5. doi: 10.3390/children9010005 (PMC8774193; doi:10.3390/children9010005)
Supplement: Supplementary file 1 [file children-09-00005-s001.zip › supplementary Figure S1.pdf]

Supplementary Materials:

- Breathe through pursed lips, as if you are whistling. Or pinch one nostril and breathe through your nose. It is harder to hyperventilate through your nose or through pursed lips because you can't move as much air.
- Slow your breathing to 1 breath every 5 seconds, or slow enough that symptoms gradually go away.
- Try belly-breathing. This fills your lungs fully, slows your breathing rate, and helps you relax.
    - Place one hand on your belly just below the ribs. Place the other hand on your chest. You can do this while standing, but it may be more comfortable while you lie on the floor with your knees bent.
    - Take a deep breath through your nose. As you breathe in, let your belly push your hand out. Keep your chest still.
    - As you breathe out through pursed lips, feel your hand go down. Use the hand on your belly to help you push all the air out. Take your time breathing out.
    - Repeat these steps 3 to 10 times. Take your time with each breath.

Figure S1. Breathing exercises. Retrieved from: Patient Care Handouts. Hyperventilation: Care Instructions. https://myhealth.alberta.ca/Health/aftercareinformation/pages/conditions.aspx?hwid=ut2508.
